# Single-cell DNA sequencing reveals order of mutational acquisition in *TRAF7/AKT1* and *TRAF7/KLF4* mutant meningiomas

**DOI:** 10.1007/s00401-022-02485-6

**Published:** 2022-08-19

**Authors:** Helin Dogan, Christina Blume, Areeba Patel, Gerhard Jungwirth, Lisa Sogerer, Miriam Ratliff, Ralf Ketter, Christel Herold-Mende, David T. W. Jones, Wolfgang Wick, Philipp Vollmuth, Klaus Zweckberger, David Reuss, Andreas von Deimling, Felix Sahm

**Affiliations:** 1 Department of Neuropathology, University Hospital Heidelberg, Heidelberg, Germany; 2 Clinical Cooperation Unit Neuropathology (B300), German Cancer Consortium (DKTK), German Cancer Research Center (DKFZ), Im Neuenheimer Feld 224, 69120 Heidelberg, Germany; 3 Faculty of Medicine, Heidelberg University, Heidelberg, Germany; 4 Faculty of Biosciences, Heidelberg University, Heidelberg, Germany; 5 Department of Neurosurgery, University Hospital Heidelberg, Heidelberg, Germany; 6 Faculty of Medicine, Technical University of Munich, Munich, Germany; 7 Department of Neurosurgery, University Hospital Mannheim, Mannheim, Germany; 8 Department of Neurosurgery, University Hospital Saarland, Homburg, Saar, Germany; 9 Hopp Children’s Cancer Center (KiTZ), Heidelberg, Germany; 10 Division of Pediatric Glioma Research, German Cancer Research Center (DKFZ), Heidelberg, Germany; 11 Clinical Cooperation Unit Neurooncology, German Consortium for Translational Cancer Research (DKTK), German Cancer Research Center (DKFZ), Heidelberg, Germany; 12 Department of Neurology and Neurooncology Program, National Center for Tumor Diseases, Heidelberg University Hospital, Heidelberg, Germany; 13 Department of Neuroradiology, Heidelberg University Hospital, Heidelberg, Germany

Most meningiomas carry mutations in the tumor suppressor neurofibromatosis gene 2 (NF2) on chromosome 22q, while *NF2*-wildtype meningiomas account for about a third of all meningiomas [4, 7]. In non-*NF2*-mutated cases, *SMO*, *POLR2A*, *PIK3CA*, *AKT1,* and *KLF4* mutations, the latter both regularly with TRAF7 mutations, have been described [1–3, 5]. TRAF7, a E3 ubiquitin ligase which promotes degradation of p53 and p65 as well as a number of oncogenic protein targets, including NEMO, c-FLIP, and c-Myb, occurs in nearly one-fourth of all meningiomas (24% in Clark et al., 30% in Reuss et al.). However, some studies may have been enriched for specific subtypes. In almost half of the cases they co-occur with AKT1 (44% in Clark et al.) or KLF4 (36% in Clark et al.), respectively [3, 8]. The combination is intriguing: *AKT1/TRAF7* mutations are associated with meningothelial histology and basal localization, while *KLF4/TRAF7* mutations are highly specific for secretory meningioma without any predominant localization. Also, *AKT1* and *KLF4* have clear hotspots, with all mutations occurring at AKT1E17K or KLF4K409Q. In contrast, *TRAF7* mutations can occur throughout the WD40 domain of the protein (Supplementary Fig. 4, online resource). The order of mutational acquisition, whether alterations in *TRAF7* or in *AKT1/KLF4* occurs first, remains elusive.

The analyses here were initiated after diagnostic work-up of the tumors of a 47-year-old male patient with two independent meningiomas having identical somatic *TRAF7* mutation N520S, but separate *AKT1* (skull base, meningothelial) and *KLF4* (convexity, secretory type) hotspot mutation (Fig. 1a). Of note, no *TRAF7* mutation was detected in germline control DNA and surgical resection was performed at the same time. Although mere coincidence cannot be ruled out, this may be caused by a mosaicism for *TRAF7* affecting arachnoidal cells, or a single ancestor of both tumors despite macroscopically separate location. Both the latter strongly suggest TRAF7 as the initiating mutation.

**Fig. 1:**
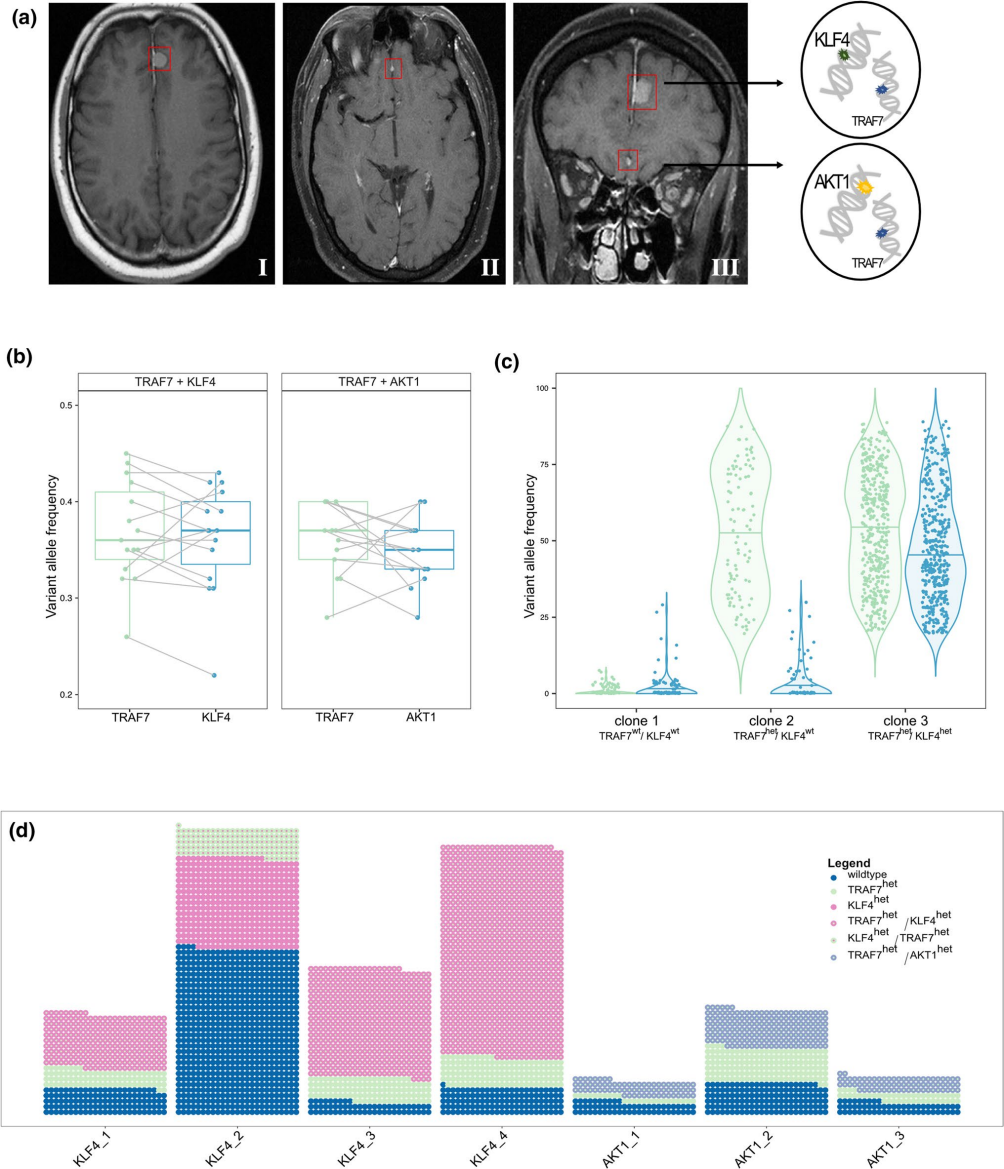
**a** Preoperative magnetic resonance imaging scans (I: axial T1-weighted gadolinium-enhanced image; II/III: axial/coronal T1-weighted fat-saturated gadolinium-enhanced image) showing two intracranial meningiomas arising from the falx cerebri, one of them left frontal parasagittal with a diameter of 12 mm [see I and III] and one of them right frontobasal with a diameter of 3 mm [see II and III]. **b** Variant allele frequencies measured from bulk sequencing data. Boxplots show the paired VAFs of TRAF7 and KLF4 on the left and TRAF7 and AKT1 on the right in 28 co-mutated meningiomas. Corresponding data from identical cases is highlighted with connecting lines. Medians: 0.37, 0.35, 0.36, 0.37. **c** Exemplary single cell clone analysis of sample KLF4_4 based on genotype clustering demonstrating allele frequency distribution within each of the three clones identified by the presence of TRAF7 N520S and KLF4 K409Q as indicated in the Supplementary Methods, online resource. **d** Overview of the distribution of mutations in seven co-mutated meningiomas. Dots represent single cells while the color indicates their mutational status. Clones are numbered according to their phylogenetic order

In an additional 28 cases of *AKT1*^*mut*^/*TRAF7*^*mut*^ (*n* = 13) and *KLF4*^*mut*^ /*TRAF7*^*mut*^ (*n* = 15) from our database we compared the variant allele frequencies (VAFs) for possible indications of heterogeneity and temporal sequence. 27/28 of the co-mutated samples were classified as WHO grade I meningiomas. 12/15 tumors of the *KLF4*^*mut*^/*TRAF7*^*mut*^ cohort were expectedly of the secretory subtype, one tumor was of transitional subtype (subtyping and grading based on the WHO classification 2016) and 2/15 were not assigned in records (no slides available for review). In line, 11/13 tumors of the *TRAF7*^*mut*^/*AKT1*^*mut*^ cohort were of the meningothelial subtype, while 2/13 were of transitional subtype. *AKT1* and *KLF4* mutations displayed clear hotspots, all occurring at AKT1E17K or KLF4K409Q. In contrast, a broad variety of *TRAF7* mutations appeared, the two most frequent locations being N520S/H/T (8/28) and K615E/T (5/28) (Supplementary Table 1, online resource).

Looking at mutational co-occurrence in bulk data, mutations assigned with higher VAFs, unless explained by copy number changes, are typically thought to be acquired earlier than those with lower VAFs. Our bulk-measured variant allele frequencies, being similar for both mutations (p-values received from two-sided Wilcoxon signed-rank test *AKT1*^*mut*^/*TRAF7*^*mut*^: p = 0.75; *KLF4*^*mut*^/*TRAF7*^*mut*^: p = 0.62) suggested no major gap between the time points of mutational acquisition (Fig. 1b, for single-cell VAFs, see Supplementary Fig. 2). However, the majority of single-mutated cases (18/33, Supplementary Methods, online resource) harbored mutations in *TRAF7* only, with fewer cases being only *KLF4* (n = 3) or only *AKT1* (n = 12) mutant.

Single-cell sequencing technologies allow more insight into the clonal architecture and complexity of thousands of individual cells. We; thus, performed amplicon-based single-cell DNA sequencing on 7 samples of *TRAF7* with *KLF4* or *AKT1*, respectively, co-mutated meningiomas. For this purpose, a custom panel covering the variants of interest and other genes relevant in CNS tumors was designed based on our custom panel for routine next-generation sequencing [6]. The panel consisted of 392 amplicons covering 28 genes and the *TERT* promoter region (Supplementary Table 2, online resource). A total of 875,000 cells were prepared resulting in a median throughput of 2315 cells per sample (interquartile range (IQR): 1622–2953) and a median coverage of 105X (IQR: 88X-122X) using the droplet-based technology of Mission Bio (Tapestri). For more details on sequencing metrics see Supplementary Table 3, online resource.

Genotype clustering analysis was performed using the Tapestri bioinformatics pipeline v2. In short, single cells were initially classified into clonal populations based on the variants known from bulk sequencing data. Only cells genotyped for both of the variants were included.

Our data revealed three subclones in each sample: one wildtype clone (potentially stroma cells), another clone carrying a mutation in *TRAF7* without any mutation in *KLF4* or *AKT1* (detected for 6/7 samples) and another clone harboring the co-mutations in *TRAF7* and *KLF4* or *AKT1* (Fig. 1c and d), for details on clone sizes (see Supplementary Table 3, online resource). This clearly indicates the *TRAF7* mutation as being acquired at an earlier stage than *KLF4* or *AKT1* in the majority of cases. Interestingly, 1/7 samples showed one clone carrying only a heterozygous *KLF4* mutation and another with the *KLF4* mutation along with the *TRAF7* mutation, both in addition to a wildtype clone.

Although single-cell DNA sequencing in particular is associated with technical challenges such as false positive variant calling and allelic dropouts, high numbers of recovered cells as well as high sequencing metrics allow conclusive information on cellular zygosity and a robust analysis of mutational acquisition. In our cohort, the single-cell data suggest, in this small series, that *TRAF7* mutation is typically acquired first, but in line with bulk data also supports that the co-mutation of *TRAF7* is still either not indispensably needed in every *KLF4* or *AKT1* mutant meningioma, or that some *TRAF7*-modifying events are not captured with the current approaches.

## Supplementary Information

Below is the link to the electronic supplementary material.

Supplementary file 1 (DOCX 53 KB)

Supplementary file 2 (TIFF 782 KB)

Supplementary file 3 (TIFF 598 KB)

Supplementary file 4 (XLSX 20 KB)

Supplementary file 5 (XLSX 69 KB)

Supplementary file 6 (XLSX 12 KB)

Supplementary file 7 (XLSX 10 KB)
