# Supplementary material for: Single-cell DNA sequencing reveals order of mutational acquisition in TRAF7/AKT1 and TRAF7/KLF4 mutant meningiomas
Source: Acta Neuropathol. 2022 Aug 19;144(4):799–802. doi: 10.1007/s00401-022-02485-6 (PMC9468091; doi:10.1007/s00401-022-02485-6)
Supplement: Supplementary file 1 — (DOCX 53 KB) [file 401_2022_2485_MOESM1_ESM.docx]

**Supplementary Material and Methods**

**Sample Selection**

Case selection was based on the database of the Dept. of Neuropathology Heidelberg, querying exonic and nonsynonymous mutations of *TRAF7, AKT1, KLF4* SNVs in meningiomas. 15 meningiomas carrying mutations in *TRAF7* and *KLF4* (*KLF4^mut^/TRAF7^mut^*) and 13 meningiomas carrying mutations in *TRAF7* along with mutations in *AKT1* (*AKT1^mut^/TRAF7^mut^*) were identified. An additional number of 33 meningiomas which only harbored one of the mutations was included: 18/33 cases harbored mutations in *TRAF7*, while the others were either only *KLF4* (n=3) or *AKT1* (n=12) mutant. Additional cryopreserved tissue of 7 samples was provided by the Dept. of Neurosurgery and Neuropathology in Heidelberg and Homburg. Of the 7 samples, 4 carried the combination *KLF4^mut^*/*TRAF7^mut^*, while 3 carried *AKT1^mut^*/*TRAF7^mut^*. Analysis was performed in accordance with local ethical regulations (2018-614N-MA, 005/2003).

**DNA methylation profiling**

Genomic DNA was extracted from formalin-fixed and paraffin-embedded (FFPE) tissue samples using the Promega Maxwell RSC DNA FFPE Kit (AS1450, Promega) on a Maxwell RSC 48 instrument (AS8500, Promega) per manufacturer’s instructions. DNA concentrations were measured on a microplate reader (FLUOStar Omega, BMG Labtech) using the Invitrogen Qubit DNA BR Assay Kit (Q32851, Thermo Fisher Scientific). DNA methylation data were generated using the Infinium MethylationEPIC (850k) BeadChip (Illumina, San Diego, USA) as previously described [2, 3]. Copy-number variation analysis was performed using the conumee Bioconductor package version 1.12.0. DNA methylation status of 10.000 CpG sites was analyzed on the current version v12b4 of the Classifier (<https://www.molecularneuropathology.org/mnp>).

**Next‑generation DNA panel sequencing and mutational analysis**

Hybrid-capture-based next-generation DNA sequencing (Agilent) was performed on a NextSeq 500 instrument (Illumina, San Diego, USA) as previously described [5] using our customized brain tumor panel covering the entire coding and selected intronic and promoter regions of 170 genes of particular relevance in central nervous system tumors. Reads were aligned against the human reference genome (GRCh37).

**Variant allele frequency calculations from bulk sequencing data**

The variant allele frequency was defined as the sum of altered forward and reverse reads divided by total reads covering the locus. Given a total sequencing depth of 3000 for a specific locus, with 1000 wildtype forward reads, 1000 wildtype reverse reads, 500 altered forward reads and 500 altered reverse reads, the variant allele frequency is calculated as follows: (500+500)/(10000+10000+500+500) = 1/3.

**Single nuclei isolation from cryopreserved tumor tissue**

Single nuclei were extracted from cryopreserved tumor tissues following a customized protocol for brain tumor nuclei isolation including previously described steps with modifications [4] [https://doi.org/10.1101/2020.10.23.351809]. Briefly, tumor content and quality of each tissue were assessed on HE stains and 150 μm slices comprising a total of 30-40 mg were cut in the same orientation. The tissue was mechanically lysed through pipetting up and down after addition of 5 ml lysis buffer (0.32 M sucrose [Sigma-Aldrich 84097], 5 mM calcium dichloride [Sigma-Aldrich 21115], 3 mM magnesium acetate [Sigma-Aldrich 63052], 2.0 mM EDTA [Invitrogen 15575-038], 0.5 mM EGTA [Alfa Aesar J61721], 10 mM Tris-HCl, pH 8.0 [Invitrogen AM98556], 1 mM DTT [Sigma-Aldrich 10197777001] and 0.1% Triton X-100 [Sigma-Aldrich 93443]). Next, the suspension was transferred into a glass douncer (Sigma-Aldrich D9063) and further lysed by douncing 15 strokes each with pestle A and B. The lysate was directly filtered through a 100 μm filter (Greiner Bio-One 542000) followed by a 40 μm filter (Greiner Bio-One 542040) into a pre-cooled and coated Falcon tube. After spinning (500g, 5 min at 4°) and washing (wash buffer: lysis buffer w/o Triton X-100 and DTT), the nuclear pellet was carefully resuspended in 100 μl wash buffer to minimize clump formation. The final nuclei quality and number was assessed on a fluorescence automated cell counter (Luna-FL, Logos Biosystems, Anyang-si, South Korea) by staining a 9 μl aliquot with Acridin Orange (AO)/ Propidium Iodide (PI) Stain (F23001-LG, biocat) at a 1:10 ratio.

**Single cell DNA sequencing library preparation and sequencing**

Nuclei concentrations were adjusted to 125,000 cells each in a total volume of 35 μl, which were loaded onto the Tapestri platform (Mission Bio, San Francisco, USA) for cell encapsulation according to Tapestri Single Cell DNA Sequencing User Guide v2. Lysis and protease digestion were then performed at 50°C for 60 minutes followed by 80°C for 10 minutes. The DNA as part of the encapsulated cell lysate was barcoded for subsequent targeted PCR amplification. For multiplex PCR, a custom panel was designed on Tapestri Designer based on COSMIC and the panel used for hybrid-capture-based next-generation DNA sequencing (Agilent) at Neuropathology, Heidelberg. The panel targets hotspot variants and prone regions across 29 genes of particular relevance in central nervous system tumors with a total of 392 amplicons and a median amplicon length of 249 bp (see Supplementary Table 2, online resource for details on panel design). Targeted PCR was performed with the following settings: 6 minutes at 98°C; 30 seconds at 95°C, 10 seconds at 72°C, 9 minutes at 61°C, 20 seconds at 72°C for 10 cycles; 30 seconds at 95°C, 10 seconds at 72°C, 9 minutes at 48°C, 20 seconds at 72°C for 10 cycles; and a final step of 2 minutes at 72°C followed by holding at 4°C. The emulsions were then broken and the PCR products collected for cleanup using AMPure XP beads (Beckman Coulter). Indices and Illumina sequencing adapters were finally added via a 10-cycle PCR reaction according to manufacturer’s instructions followed by another cleanup using AMPure XP beads. Library size was determined using the D1000 Screen Tape on the TapeStation 4200 platform (Agilent, California, USA). Concentrations were determined on the Qubit 4 Fluorometer (Thermo Fisher Scientific, Massachusetts, USA) with the Invitrogen Qubit DNA HS Assay Kit (Thermo Fisher Scientific, Q32854). Excessive primer dimers were removed when necessary, according to the manufacturer’s recommendations. The final libraries were pooled and loaded on a NovaSeq 6000 instrument (Illumina, San Diego, USA), using the S1 flow cell and the 300 cycles kit for a 150 bp paired-end run. For sequencing and sample quality metrics see Supplementary Table 4 and Supplementary Figure 3, online resource.

**Single cell data processing**

Sequencing data was processed using the Tapestri bioinformatics pipeline v2 for adapter trimming, alignment to reference genome hg19, barcode assignment to single cells, genotyping and variant calling using a GATK based algorithm. Loom files generated by the Tapestri pipeline were then loaded into the Tapestri Insight v2.2 software for filtering and further analysis. A whitelist including the variants of interest in *TRAF7*, *KLF4* and *AKT1*, was uploaded and all other variants were removed. Recommended filtering parameters for selecting high quality genotype and variant calls were used for the *KLF4^mut^/TRAF7^mut^* samples, while they needed to be adjusted for the *AKT1^mut^/TRAF7^mut^* samples due to lower amplicon performance: genotype quality score < 5 (more permissive), read depth < 0 reads (more permissive), variant allele-frequency < 20 % (default), variant genotyped in < 20 % of the cells (more permissive), cells with < 0% of the genotypes present (more permissive), and variants mutated in < 1 % (default). For more detail on the filtering parameters please see <https://support.missionbio.com/hc/en-us/articles/360042326414>.

**Single cell data analysis: Identifying ADOs and order of mutational acquisition**

Single cells were initially classified into clonal populations including zygosity information based on the variants known from bulk sequencing data. Only cells genotyped for both of the variants were included. All clones were then assessed for allelic dropout (ADO) events thereby identifying true clones and their evolutionary trajectory. In short, ADO events occur in a subset of the cells at a site which is known to be heterozygous, stochastically affecting the wildtype or the alternate allele and can be identified as previously described by Alberti-Servera et al. [1]. Following this, an ADO event is indicated by 1) the number of wildtype and homozygous cells being similar and 2) the read quality (RQ) being decreased in those cells compared to their heterozygous parent clone since the RQ is a metric which impacts the ability to robustly call genotypes. Despite lowering filters for *AKT1^mut^/TRAF7^mut^* samples, quality metrics remained high enough for clear ADO identification (Supplementary Fig. 1, online resource). Cells which were identified as affected by allelic dropout were assigned to their maximum likelihood genotype. Figures were generated using custom pipelines in R studio version 3.6.3 using filtered data exported from Tapestri Insights v2.2.

**Statistics**

Statistical tests were performed using two-sided Wilcoxon signed-rank test for matched samples (*TRAF7*+*AKT1* and *TRAF7*+*KLF4*). P-values <0.05 were considered statistically significant. Plots were created using R package ggplot2 version 3.3.0 and tidyverse version 1.3.0 (<https://ggplot2.tidyverse.org>). All analysis was performed on RStudio Version 3.6.3. To evaluate the distribution of the calculated allele frequencies, box plots, violin plots and dot plots were created for each mutation grouped by the both cohorts *TRAF7*+*AKT1* and *TRAF7*+*KLF4* using ggplot2 in R. The plots were generated by default with whiskers extending up to 1.5x interquartile range (IQR) and data further than 1.5x IQR being considered as ‘outliers’ and therefore plotted individually.

**References**

1 Alberti-Servera L, Demeyer S, Govaerts I, Swings T, De Bie J, Gielen O, Brociner M, Michaux L, Maertens J, Uyttebroeck Aet al (2021) Single-cell DNA amplicon sequencing reveals clonal heterogeneity and evolution in T-cell acute lymphoblastic leukemia. Blood 137: 801-811 Doi 10.1182/blood.2020006996

2 Capper D, Jones DTW, Sill M, Hovestadt V, Schrimpf D, Sturm D, Koelsche C, Sahm F, Chavez L, Reuss DEet al (2018) DNA methylation-based classification of central nervous system tumours. Nature 555: 469-474 Doi 10.1038/nature26000

3 Capper D, Stichel D, Sahm F, Jones DTW, Schrimpf D, Sill M, Schmid S, Hovestadt V, Reuss DE, Koelsche Cet al (2018) Practical implementation of DNA methylation and copy-number-based CNS tumor diagnostics: the Heidelberg experience. Acta Neuropathol 136: 181-210 Doi 10.1007/s00401-018-1879-y

4 Narayanan A, Blanco-Carmona E, Demirdizen E, Sun X, Herold-Mende C, Schlesner M, Turcan S (2020) Nuclei Isolation from Fresh Frozen Brain Tumors for Single-Nucleus RNA-seq and ATAC-seq. J Vis Exp: Doi 10.3791/61542

5 Sahm F, Schrimpf D, Jones DT, Meyer J, Kratz A, Reuss D, Capper D, Koelsche C, Korshunov A, Wiestler Bet al (2016) Next-generation sequencing in routine brain tumor diagnostics enables an integrated diagnosis and identifies actionable targets. Acta Neuropathol 131: 903-910 Doi 10.1007/s00401-015-1519-8
