# Supplementary figures and images for: Single-cell DNA sequencing reveals order of mutational acquisition in TRAF7/AKT1 and TRAF7/KLF4 mutant meningiomas
Source: Acta Neuropathol. 2022 Aug 19;144(4):799–802. doi: 10.1007/s00401-022-02485-6 (PMC9468091; doi:10.1007/s00401-022-02485-6)

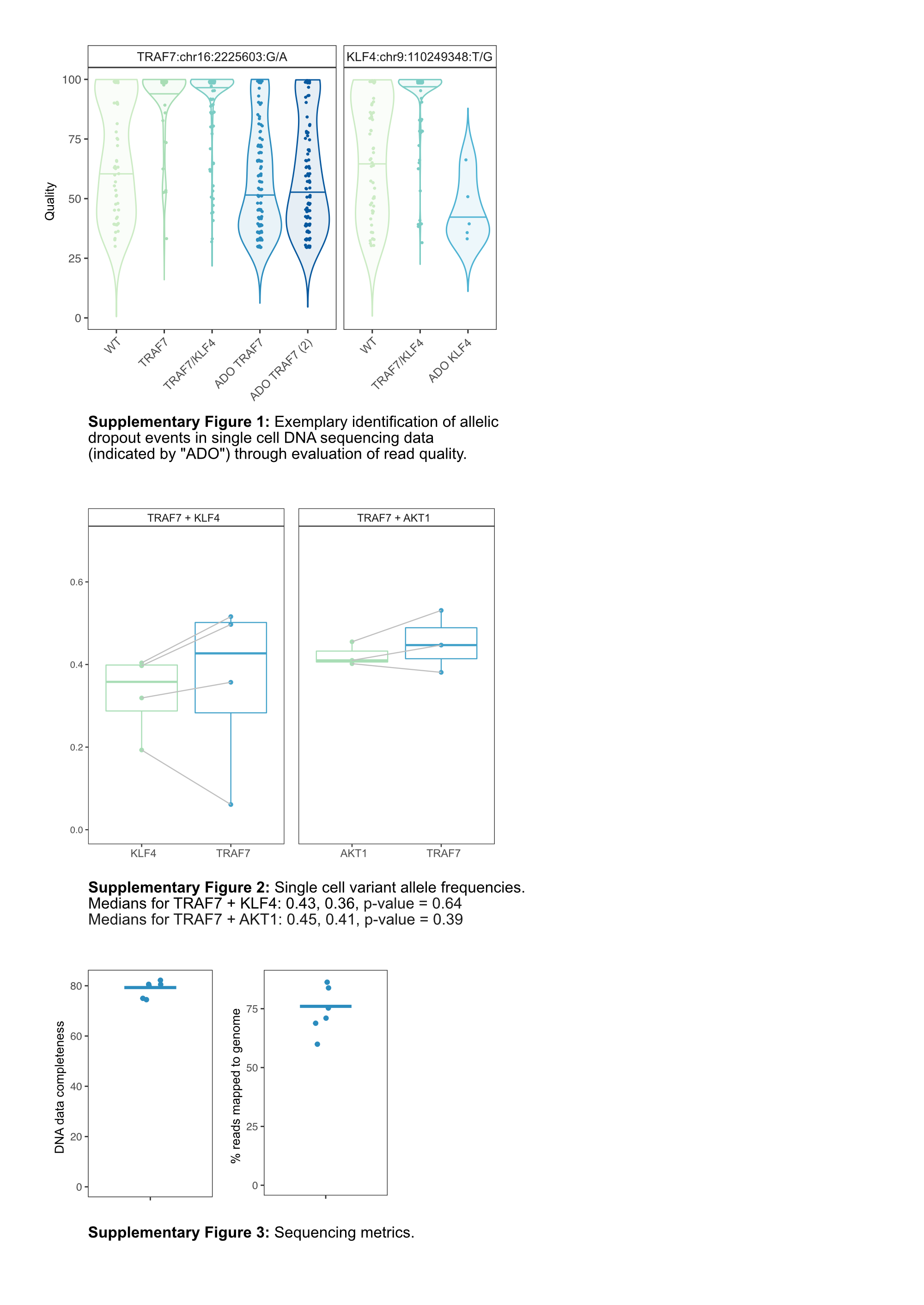

Supplement: Supplementary file 2 — (TIFF 782 KB) [file 401_2022_2485_MOESM2_ESM.tiff]

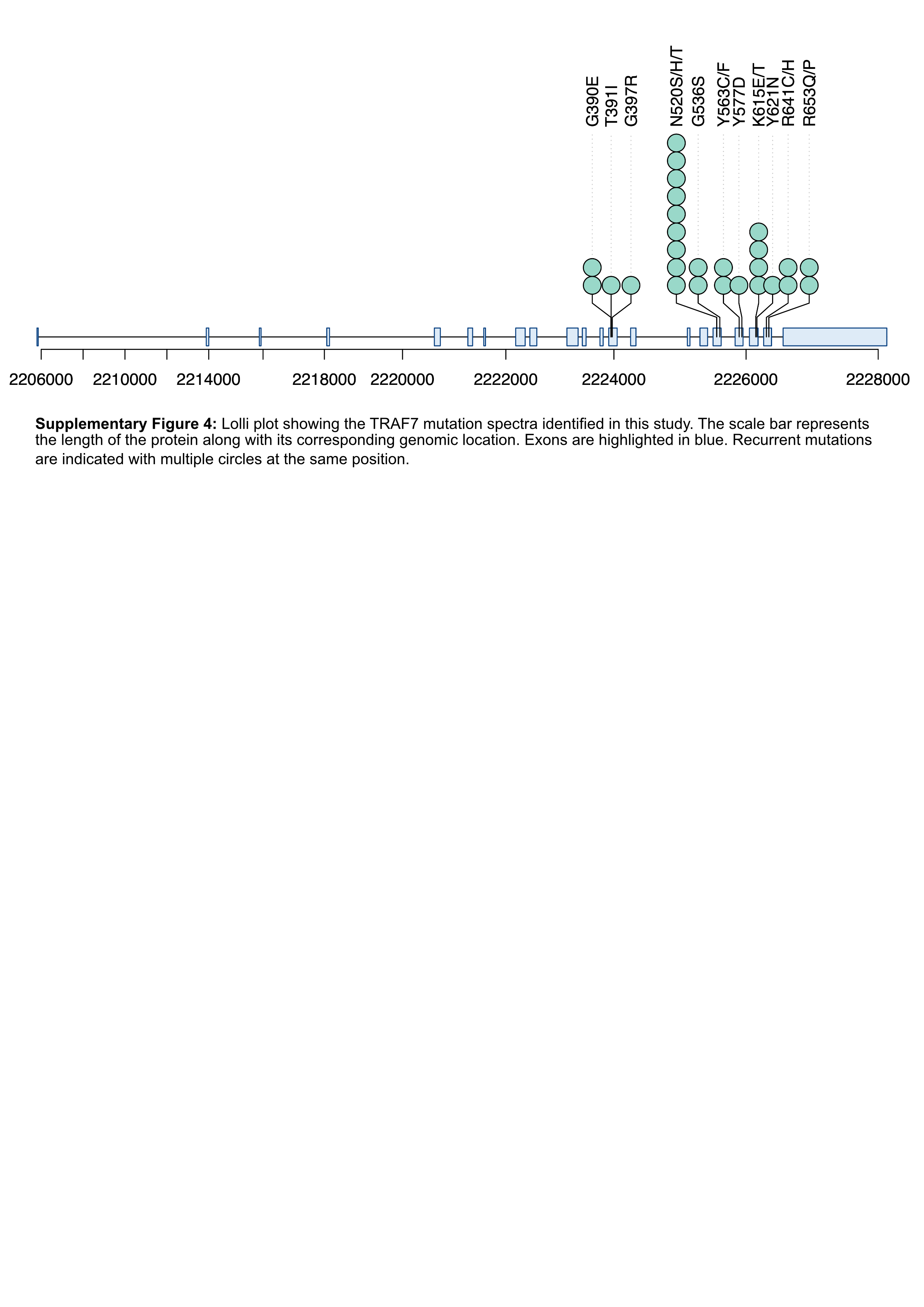

Supplement: Supplementary file 3 — (TIFF 598 KB) [file 401_2022_2485_MOESM3_ESM.tiff]
